# Nocturnal Pain Crises in an Adult with Sickle Cell Disease

**DOI:** 10.7759/cureus.57462

**Published:** 2024-04-02

**Authors:** Konstantina Papadopoulou, Efthymia Papadopoulou, Christoforos Proimos, Zacharo Sachla, Stavros Tryfon

**Affiliations:** 1 Internal Medicine Department, "G. Papanikolaou" General Hospital of Thessaloniki, Thessaloniki, GRC; 2 Pulmonology Department, "G. Papanikolaou" General Hospital of Thessaloniki, Thessaloniki, GRC

**Keywords:** continuous positive airway pressure (cpap), sleep-disordered breathing, obstructive sleep apnea (osa), drepanocytosis, sickle cell disease (scd)

## Abstract

Sickle cell disease is the most common genetic hemoglobinopathy worldwide, characterized by a single-nucleotide mutation that predisposes to hemoglobin polymerization and erythrocyte sickling in hypoxic states. This report describes a 62-year-old male obese patient with a history of sickle cell disease, who presented with worsening nocturnal pain crises without any apparent triggering factor. A thorough evaluation at the outpatient department revealed obstructive sleep apnea. Airway obstruction or decreased respiratory effort during sleep may induce hypoventilation and hypoxia in the context of sleep-disordered breathing, with severe cardiopulmonary complications. Sleep-disordered breathing is considered common in children with sickle cell disease, but the prevalence in adults has not been sufficiently documented. Our patient responded favorably to treatment with continuous positive airway pressure during sleep, showing complete resolution of his symptoms. Timely diagnosis and management are fundamental to improve outcomes and prevent severe complications.

## Introduction

Drepanocytosis or sickle cell disease (SCD) is the most common genetic hemoglobinopathy worldwide, inherited as an autosomal recessive disorder. It is characterized by a single-nucleotide mutation in the β-globin chain of hemoglobin, that predisposes to hemoglobin polymerization and erythrocyte sickling in hypoxic states [1]. Manifestations comprise anemia, hemolysis, acute and chronic microvascular vaso-occlusive events, while chronic cardiopulmonary complications are major contributors to mortality [1]. Comorbidities should be carefully monitored to improve disease prognosis.

Airway obstruction or decreased respiratory effort during sleep may induce hypoventilation and hypoxia in the context of sleep-disordered breathing, with severe cardiopulmonary complications. Obstructive sleep apnea is highly prevalent in the general population, amounting to 9-37% in males and 4-50% in females [2]. Risk factors comprise mostly adenotonsillar hypertrophy, increased neck circumference, and obesity [2]. As the patients may not complain of symptoms, high clinical awareness is fundamental to timely diagnosis and treatment.

Sleep-disordered breathing is common in the pediatric population with SCD [3,4], but the prevalence has been explored only in limited underpowered studies in adults [5-7], resulting in a lack of clinical recognition. This case report describes an adult patient with SCD, who presented with worsening nocturnal pain crises without any apparent triggering factor. A thorough evaluation at the outpatient department revealed obstructive sleep apnea. As the patient did not report symptoms related to obstructive sleep apnea, aggravation of SCD pain crises rendered the diagnostic approach challenging. 

This article was previously presented as a meeting abstract at the 6th Panhellenic Conference of Chest Diseases on May 26, 2023.

## Case presentation

A 62-year-old male presented at the outpatient department with intermittent nocturnal chest pain, usually concurring with hand and foot pain at arousals from sleep. These episodes did not respond to analgesics but resolved in the daytime. There was a gradual worsening within one year. His medical history included SCD since childhood with no need for treatment with hydroxyurea and blood transfusions, other than occasional analgesics in cold weather. He was a former smoker with 40 pack-years smoking history.

**Figure 1:**
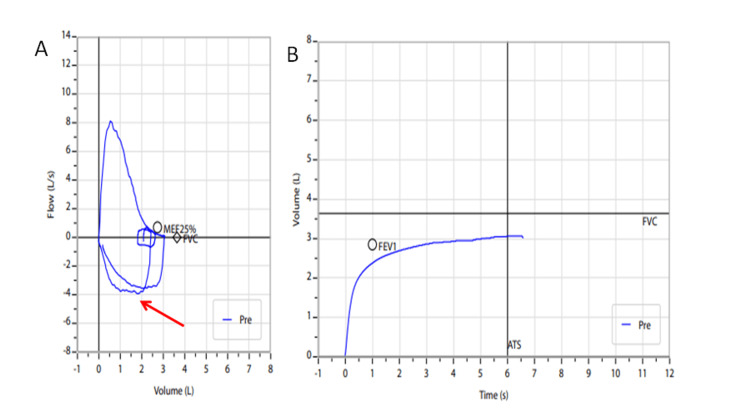
Spirometry revealed normal values, but the inspiratory flow-volume curve appeared flattened and "wavy" (red arrow), indicating a variable extra-thoracic upper airway obstruction. A: Flow-volume curve; B: Volume-Time curve. Pre: pre-bronchodilation spirometry; MEF25%: maximal expiratory flow at 25% of the forced vital capacity; FVC: forced vital capacity; FEV1: forced expiratory volume in the first second; ATS: American Thoracic Society spirometry criteria.

The patient was obese with a body mass index of 35.1 kg/m^2^; his body weight had reportedly increased within the past year. His vital signs, chest X-ray, and blood tests were normal, apart from stable moderate anemia (hemoglobin 9.4 g/dL) consistent with his previous measurements. A cardiology assessment did not reveal any notable pathology. In view of his smoking history, spirometry was performed, with normal values (forced vital capacity=89% of the predicted value, forced expiratory volume in the first second=94% of the predicted value, and Tiffeneau-Pinelli index=82.2%). However, the inspiratory flow-volume curve appeared flattened and “wavy” (Figure 1), indicating a variable extra-thoracic upper airway obstruction, such as vocal cord and rhinopharyngeal edema.

Upon further questioning, his partner confirmed uneasy sleep with snoring, but the patient did not complain of any sleep-related symptoms himself. Considering potential symptom underestimation by the patient, we used the Epworth Sleepiness Scale as a validated measure to assess daytime sleepiness. He scored 14 points, compatible with excessive daytime sleepiness.

**Figure 2:**
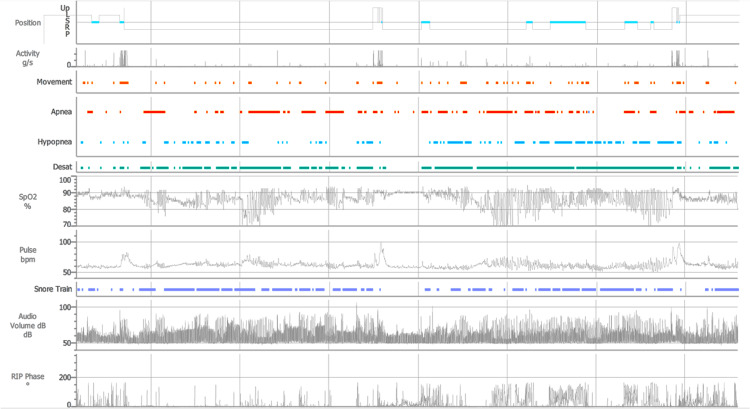
Polysomnography was compatible with severe obstructive sleep apnea. L: left; S: supine; R: right; P: prone; Desat: desaturation events; SpO2: oxygen saturation measured by pulse oximetry; bpm: beats per minute; dB: decibel; RIP: respiratory inductance plethysmography.

Οbstructive sleep apnea-hypopnea syndrome was suspected and the patient underwent a polysomnography study, which demonstrated an apnea-hypopnea index (AHI) of 55.1 events per hour, an oxygen desaturation index (ODI) of 53 per hour, desaturation (SpO2<90%) for 289.7 minutes amounting to 66.5% of total sleep time, an average SpO2 value of 87.6% and a lowest SpO2 value of 66% (Figure 2). These findings established the diagnosis of severe obstructive sleep apnea, without hypercapnia.

The patient was strongly encouraged to lose weight following specific dietary instructions and an exercise program, as well as to avoid sedatives and alcohol before sleep. Continuous positive airway pressure (CPAP) treatment at sleep was initiated following titration. At follow-up one week later, the patient’s symptoms and pain crises had significantly subsided. During six months of follow-up, he showed appropriate compliance with CPAP treatment and total resolution of pain crises.

## Discussion

This case highlights a challenging clinical presentation of SCD complicated by sleep-disordered breathing in an adult patient, where hypoxia during obstructive sleep apnea events induced SCD pain crises.

Several studies have shown a higher prevalence of sleep-disordered breathing in children with SCD [3] compared to the general pediatric population [4], but the prevalence in adults has not been sufficiently documented. In a small study of 32 adults with SCD and sleep disturbances or high Epworth Sleepiness Scale score, polysomnography revealed sleep apnea hypopnea syndrome (AHI ≥ 5) in 44% of SCD patients, with predominantly central apnea in only one [5]. Another small study estimated a prevalence of 50% among young adults with SCD [6]. A significant difference has been reported compared to healthy volunteers (67% versus 22%) [7].

Adenotonsillar hypertrophy is noted in higher prevalence in children with SCD [8], potentially secondary to splenic infarction or reactive lymph node enlargement due to recurrent infections. Our patient was obese and had increased neck circumference, which is a well-known risk factor for obstructive sleep apnea. Chronic opioid use or comorbid congestive heart failure may also predispose to central sleep apnea in adults with SCD [9], which was not observed in our patient.

SCD and sleep-disordered breathing promote oxidative stress, chronic inflammation, and autonomic dysfunction, involving common pathophysiologic pathways related to hypoxia and inflammatory cascades [10-12]. Intermittent nocturnal hypoxia and re-oxygenation induce oxidative stress promoting tissue damage and affecting the bioavailability of nitric oxide (NO), with subsequent endothelial dysfunction, aggregation of sickled erythrocytes and, ultimately, vaso-occlusive events [10]. Transient hypoxia also induces hyperactive autonomic nervous system responses in sleep-disordered breathing [10], which may result in peripheral vasoconstriction manifesting as acute pain crises in SCD [13].

Sleep-disordered breathing has been associated with acute and chronic complications in children with SCD. These include vaso-occlusive events, pulmonary hypertension, cardiovascular abnormalities and neurologic deficits [14]. Literature data on adults with SCD are scarce, but some small studies showed impaired exercise capacity, diastolic cardiac function, and quality of life [6], as well as more pain crises leading to hospitalization than in counterparts without sleep-disordered breathing [7]. Our patient presented with nocturnal pain crises, without cardiovascular impairment. Given the generally mild course of SCD prior to body weight gain, the appropriate treatment of obstructive sleep apnea led to the complete resolution of his symptoms and hopefully to the prevention of cardiovascular complications. 

The 2019 American Society of Hematology Cardiopulmonary and Renal Guidelines for SCD [15] recommend meticulous screening for signs and symptoms of sleep-disordered breathing with a low threshold to polysomnography, as typical symptoms may be absent [9]. In consideration of the association between pulmonary hypertension and obstructive sleep apnea, the American Thoracic Society Clinical Practice Guidelines for Diagnosis and Treatment of Pulmonary Hypertension in SCD suggest polysomnography for SCD patients with pulmonary hypertension [16].

Treatment with hydroxyurea may alleviate symptoms of sleep-disordered breathing and enhance nocturnal and daytime oxygen saturation in patients with SCD [9,17,18]. Phase 1 randomized trials have found auto-adjusting CPAP to be feasible and safe both in children and adults with SCD and sleep-disordered breathing [19,20]. Our patient responded favorably to CPAP treatment for obstructive sleep apnea, showing complete resolution of his symptoms. 

## Conclusions

We presented a challenging clinical case, where hypoxia related to obstructive sleep apnea induced pain crises in an adult SCD patient without other disease manifestations. The patient-reported gradual increase in body weight and the variable extra-thoracic upper airway obstruction apparent in the spirometry flow-volume curve raised suspicion of obstructive sleep apnea, although the patient did not complain of any related symptoms. Treatment with CPAP during sleep led to complete resolution of his pain crises, confirming the causative role of desaturation events during sleep.

Meticulous screening for signs and symptoms of sleep-disordered breathing is fundamental to timely diagnosis and prevention of major complications in patients with SCD. Considering the high prevalence of sleep-disordered breathing, compatible clinical features should prompt clinical suspicion in patients with decompensation of other chronic disorders, even in the absence of reported symptoms.
